# Re-irradiation Using Stereotactic Radiotherapy: A Bibliometric Analysis of Research Trends

**DOI:** 10.7759/cureus.39600

**Published:** 2023-05-28

**Authors:** Ahamed Badusha Mohamed Yoosuf, Muhammad Ajmal Khan, Mohd Zahri Abdul Aziz, Syahir Mansor, Gokula Kumar Appalanaido, Salem Alshehri, Mamdouh Alqathami

**Affiliations:** 1 Oncology, King Abdullah International Medical Research Center, Riyadh, SAU; 2 Department of Radiation Oncology, Ministry of National Guard-Health Affairs, Riyadh, SAU; 3 Library and Health Science, Imam Abdulrehman Bin Faisal University, Dammam, SAU; 4 Advanced Management of Liver Malignancies Program, Universiti Sains Malaysia/Advanced Medical and Dental Institute, Penang, MYS; 5 Nuclear Medicine Unit, Pusat Perubatan Universiti Sains Malaysia/Advanced Medical and Dental Institute, Penang, MYS; 6 Radiotherapy Unit, Pusat Perubatan Universiti Sains Malaysia/Advanced Medical and Dental Institute, Penang, MYS; 7 Medical Physics, King Saud Bin Abdulaziz University for Health Sciences, Riyadh, SAU

**Keywords:** quality of life, radiotherapy toxicity, stereotactic radiotherapy (srt), stereotactic ablative radiotherapy fractionation, re-irradiation

## Abstract

The objective of this research is to conduct a comprehensive bibliometric analysis using the Web of Science Core Collection (WoSCC) to examine the current research topics and trends pertaining to stereotactic-based re-irradiation.

A bibliometric search was conducted for re-irradiation-related literature published in English from the WoSCC database from 1991 to 2022, using VOSviewer to visualize the results. The extracted information comprises the publication year, overall citation count, average citation rate, keywords, and research domains. We conducted a literature review to identify trends in research on re-irradiation.

A total of 19,891 citations were found in 924 qualifying papers that came from 48 different nations. The number of publications and citations has grown steadily since 2008 with the highest number of publications in the year 2018. Similarly, a substantial increase in the number of citations has increased since 2004 and the citation growth rate has been positive between 2004 and 2019 with a peak in 2013. The top authorship patterns were six authors (111 publications and 2498 citations), whereas the highest number of citations per publication was attained with an authorship pattern of 17 authors (C/P = 41.1). The collaboration patterns analysis showed that the largest proportion of publications emanated from the United States with 363 publications (30.9%), followed by Germany with 102 publications (8.7%), and France with 92 publications (7.8%). The majority of the analyzed studies were focused on the brain (30%), head and neck (13%), lung (12%), and spine (10%) and there have been emerging studies on the use of re-irradiation for lung, prostate, pelvic and liver utilizing stereotactic radiotherapy.

The main areas of interest have changed over time and are now based on a multidisciplinary approach that integrates advanced imaging techniques, stereotactic treatment delivery, the toxicity of organs at risk, quality of life, and treatment outcomes.

## Introduction and background

Re-irradiation is used to treat recurrent or residual tumors following prior radiotherapy. In recent decades, advancements in cancer detection, staging, and management have resulted in improved disease outcomes and higher survival rates [1]. In comparison to three or four decades ago, patients receiving current appropriate care are expected to have an improved quality of life [1,2]. The issue of a localized disease recurrence in an otherwise healthy patient necessitating extra modes of treatment measures for local control and palliation has come into focus as a result of the increased control rate and survival. To maximize response and survival, current therapeutic approaches place a greater emphasis on organs at risk and their function. The increased use of innovative methods and technology, particularly in radiotherapy, has led to better normal tissue sparing, which has improved the quality of life. Stereotactic-based re-irradiation has emerged as a viable treatment option for patients with loco-regional recurrence, with few other treatment options. This treatment approach delivers high doses of radiation to the tumor with pinpoint accuracy while sparing the healthy tissue. It has been used to treat patients who have failed prior radiation therapy, as well as those who are not surgical candidates. More retrospective clinical data have been reported and published in the last two decades with generally good efficacy and acceptable toxicity [3-8]. As a result, re-irradiation using stereotactic radiotherapy is considered a more accurate treatment, delivering a highly biologically effective dose (BED) to tumors while minimizing the dose absorbed by normal tissues, potentially reducing radiotherapy’s toxicity and side effects.

The bibliometric analysis emphasizes the key studies and topics that have influenced the management and understanding of a disease of interest and uses citation data to evaluate an article's academic effect in its chosen subject [9,10]. The bibliometric analysis enables researchers to examine the most recent findings and familiarize themselves with the areas of research that are most important. It is a very helpful tool for evaluating academic ability within a certain academic topic that employs citation patterns and frequency as objective indicators [11,12]. Additionally, a specific academic community's most significant journals, authors, contributing institutions, and nations can be identified using article citation counts [13].

Studies on re-irradiation using stereotactic radiotherapy have increasingly been published for malignancies in different regions [3-8]. The bibliometric analysis of the literature, however, is limited. In this bibliometric analysis, we aim to examine the publication patterns, research trends, keywords, countries, journals, collaboration network, and citation impact of research on re-irradiation using stereotactic radiotherapy from 1990 to 2022 using the Web of Science. Further, we wish to present an overview of the literature published in the last two decades for different tumor sites that have used re-irradiation.

## Review

Materials and methods

Data Source and Search Strategy

The dataset used in this work was retrieved from Web of Science Core Collection (WoSCC). The WoSCC is the largest database of cited scientific publications in the world, and it can be used to assess the academic significance of works on a given topic. A comprehensive search of WoSCC was performed using the strategy as follows: (TS=(reirradiation OR re-irradiation) AND TS=(stereotactic* OR SBRT OR SABR)) AND TS=(radiosurgery OR radiotherapy OR "radiation therapy"), where TS refers to “topic”. The search fields comprised the article title, abstract, and keywords. As a quantity indicator, the results were refined to include manuscripts written in English and published between 1st January 1991 and 31st December 2022. Further, we assessed the influence of the research on the scientific community using the Science Citation Index Impact Factor as a quality indicator.

Original studies, abstracts, and reviews were the only article categories considered; editorial content, letters, corrections, and retractions were not included. Cases relating to each of the chosen documents were constructed in a bibliographic data frame. Every record included bibliographic information on the author, their affiliation, the title, an abstract, keywords, the name of the journal, the year of publication, the volume, issue, page, publisher, country, and the total number of citations. "Article proceedings papers" pertain to individual articles that have undergone a peer-review process and are presented at conferences or symposiums. These papers encompass original research findings, methodologies, and results. They are typically published within conference proceedings. On the other hand, "proceedings papers" encompass the collective compilation of papers presented at a conference or symposium. These papers may encompass various types of content, including research articles, review articles, case studies, and technical reports. 

The strength of bibliometric analysis revolves around its ability to provide powerful quantitative and qualitative indicators of the title under study. The total number of publications (TP) is an important quantitative index to measure publication growth over the years. Other indices such as total citation (TC), non-cited publications (NCP), cited publications (CP) and citation per publication (C/P) contribute to the qualitative assessment in bibliometric analysis [14-16].

The key data about data collection (annual scientific production, average number of citations per year), sources (most significant most referenced, as well as dynamics), researchers (most relevant authors and associations, corresponding author's nation, nation-specific production), and reports (most worldwide cited documents, the most frequently used keywords, words dynamics) were presented in the summary function in order to highlight the key findings. The annual growth rate was used to describe the ratio of scientific production across time.

The most relevant countries were ranked by the country of corresponding authors. Country links were used to indicate author occurrences in national scholarly articles (the number of documents indicates author occurrences by country). The degree of international cooperation for a certain nation was measured by the proportion of papers with at least one co-author who was employed abroad as opposed to the corresponding authors [17]. Based on authorship associations, the most productive countries were determined. Correspondence analysis and clustering techniques were used to find common terms and author/institution relationships in research topics. A thematic map was generated from the keyword network using a clustering algorithm.

Data Analysis

Bibliographic analysis tools were used to obtain meaningful data [18]. MS Excel (V16.0) was used to perform basic functions and plot publication and citation trends. VOS Viewer (version 1.6.15) was used for data visualization, including journals, researchers, individual publications, citations, bibliographic linkage, co-citation, or co-authorship relationships. The whole process was verified by two authors to ensure data accuracy.

Results

Publications and Document Types

A total of 924 publications were identified, with an average of 30 publications per year. Table 1 displays the distribution of publications, indicating that the majority consisted of research articles (76.6%), followed by review articles (19.6%), article proceedings papers (3.0%), and proceedings papers (0.5%). The total number of citations for the publications was 19,891, with an average citation rate of 22.6 citations per publication. In total, 10.4% were NCP and 89.6% were CP. The highest citation per publication was achieved for a proceeding paper (C/P = 50.3). Furthermore, of the studies, 299 (32.3%) were retrospective and 96 (21.2%) were prospective.

**Table 1:** Publication details and their document types from 1990 to 2022. NCP – non-cited publications; CP – cited publications; TP – total publications; TC – total citations; C/P – citations per publication; GTP – grand total publications; S_year – start year; E_year – end year

Document Type	NCP	CP	TP	TC	C/P	% of GTP	S_year	E_year
Article	75	636	711	15150	21.31	76.62	1991	2022
Review	17	166	183	3332	18.21	19.59	2001	2022
Article; Proceedings Paper	3	25	28	1408	50.29	3.03	2000	2021
Proceedings Paper	1	1	2	1	0.5	0.22	2002	2017
GTP	96	828	924	19891				

Annual Publication Growth and Authorship Pattern

**Figure 1:**
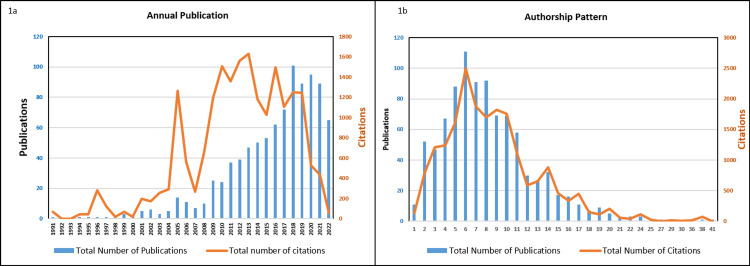
Illustration of annual publication growth (A) and authorship pattern (B) from 1991 to 2022.

Based on WoSCC, Figure 1A demonstrates the annual publishing and citation structures of re-irradiation using stereotactic radiotherapy. A total of 11 publications (1.2%) and 671 citations (3.4%) were produced between 1990 and 2000. Similarly, 110 publications (12.0%)/6363 citations (32.0%) and 799 publications (86.8%)/12856 citations (64.6%) were produced between 2001-2010 and 2011-2022, respectively. The number of publications and citations has grown steadily since 2008 with the highest number of publications in the year 2018. Likewise, a substantial increase in the number of citations has increased since 2004, and the citation growth rate has been positive between 2004 and 2019 with the highest peak in 2013 (1629 citations) as shown in Figure 1A. Publication trends indicate that research on reirradiation using stereotactic radiotherapy has increased over time. 

Figure 1B depicts the authorship pattern of publications from 1990 to 2022 in the field of re-irradiation using stereotactic radiotherapy. The top authorship patterns were six authors (111 publications and 2498 citations), eight authors (92 publications and 1698 citations), and seven authors (91 publications and 1880 citations). The authorship pattern varied between 11 publications with one author and one publication with 41 authors. The highest citations per publication were attained with an authorship pattern of 17 authors (C/P=41.1), 20 authors (C/P=39.8), and 24 authors (C/P=38.3).

Author’s Productivity

Table 2 illustrates the top 20 authors with the highest number of publications and citations in the field of re-irradiation using stereotactic radiotherapy. The total number of publications varied between 13 and 40 and citations 179 to 1566, respectively. Combs SE has the highest number of publications (n=28) as the first author (n=18) with 1239 citations. The highest citations (n=1566) were achieved by the author Debus J.

**Table 2:** Authors with the highest number of publications and citations. TP – total publications; TC – total citations

Author	Author's Publications			TP	TC	Year	
	Single	first	other			Start	End
Heron, DE		2	38	40	1090	2002	2021
Sahgal, A		3	26	29	886	2009	2022
Combs, SE		18	10	28	1239	2005	2022
Debus, J			27	27	1566	2002	2022
Ferris, RL			21	21	771	2009	2020
Vargo, JA		7	13	20	493	2012	2021
Clump, DA			19	19	339	2013	2021
Cengiz, M		1	16	17	291	2011	2022
Nieder, C	1	10	5	16	670	2005	2019
Yamada, Y			16	16	602	2011	2020
Yazici, G		3	13	16	269	2011	2022
Guckenberger, M		1	14	15	311	2011	2021
Lartigau, E		2	13	15	320	2007	2019
Noel, G		5	10	15	212	2001	2022
Ozyigit, G		4	10	14	264	2011	2022
Yamazaki, H		10	4	14	262	2011	2022
Burton, SA			13	13	309	2011	2019
Jereczek-Fossa, BA		4	9	13	370	2008	2021
Lacornerie, T		1	12	13	460	2007	2021
Phan, J		1	12	13	179	2016	2021

Keywords and Co-occurrence

**Figure 2:**
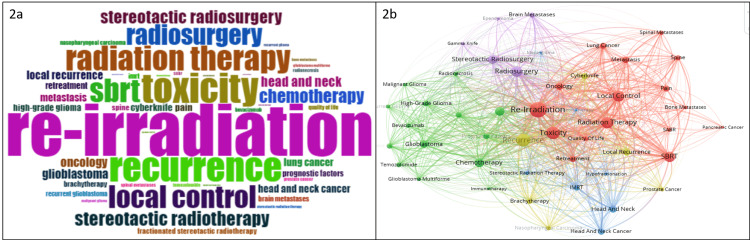
Illustration of top keywords (A) and and overly visualization of co-occurrence analysis visualization of keywords in WoSCC (B). WoSCC - Web of Science Core Collection

Figure 2A illustrates the top keywords (n=50) identified in this study used by most authors in the field of re-irradiation. Based on the findings depicted in Figure 2A, it is evident that the keywords "re-irradiation," "toxicity," "recurrence," "radiation therapy," and "local control" are the most commonly utilized terms in this field of study. Keyword combinations are used in the systematic review protocol to give a comprehensive overview of research trends. The visualization of the frequently used keywords and their correlation using the VOS viewer is shown in Figure 2B. The network includes five clusters and 48 nodes, respectively. The representation in Figure 2B presents a visual analysis of the keywords provided by the authors, where the size of each node corresponds to its frequency of repetition. Links between nodes indicate cooccurrences in the same article and the color of a node signifies the cluster it belongs to, and different clusters are characterized by different colors. The shorter the spacing between keywords, the more often the keywords will appear together. This demonstrates that the domains of "re-irradiation" and "toxicity" span the biggest and most evident area using rigorous bibliometric metrics.

**Figure 3:**
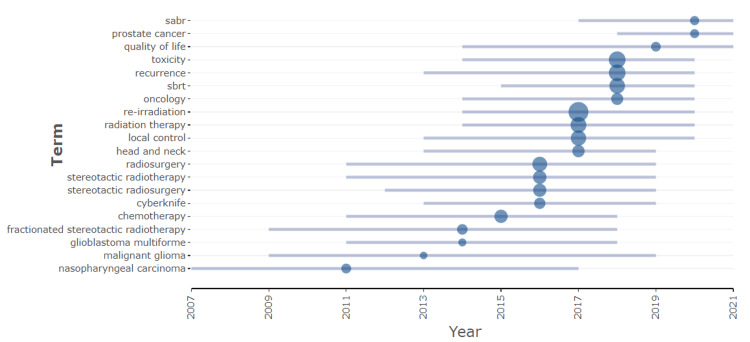
Prevalence of keywords over time and the bubble indicates the year in which it was mostly used in the literature. SBRT – stereotactic body radiotherapy; SABR – stereotactic ablative radiotherapy

Figure 3 displays the prominent keywords over time, where the bubbles represent the respective years in which they were predominantly utilized in the literature. Keywords of nasopharyngeal carcinoma, malignant glioma, and fractionated stereotactic radiotherapy were used in the early 2000 whereas keywords such as radiosurgery and stereotactic radiotherapy emerged post-2010. The keywords stereotactic body radiotherapy (SBRT), prostate, quality of life, toxicity, and stereotactic ablative radiotherapy (SABR) are emerging topics in the last few years. This indicates the research trends in the field of re-irradiation and advancement in technology that is used.

The Geographical Contribution of Publications

Table 3 shows the overall publication distributions from the top 20 countries. Forty-eight different countries produced 924 publications in the field of re-irradiations. The collaboration patterns analysis showed that the United States was the most productive country, with 363 publications (30.9%), followed by Germany with 102 publications (8.7%), and France with 92 publications (7.8%). Further, the United States contributed the most number of citations (33.9%), followed by Germany (13.8%) and France (4.8%), respectively. The citation per publication was highest in the Netherlands (C/P=42.9) followed by Germany (C/P=36.4) and Canada (C/P=26.1).

**Table 3:** Top 20 countries and their publication trend in the field of re-irradiation using stereotactic radiotherapy. NCP – non-cited publications; CP – cited publications; TP – total publications; TC – total citations; C/P – citations per publication; S_year – start year and E_year – end year

COUNTRY	NCP	CP	TP	TC	C/P	% of Total	S_year	E_year
USA	27	336	363	9136	25.17	28.79	1991	2022
GERMANY	6	96	102	3712	36.39	8.09	2002	2022
FRANCE	9	83	92	1295	14.08	7.3	2001	2022
ITALY	6	79	85	1739	20.46	6.74	1999	2022
CANADA	5	71	76	1984	26.11	6.03	1994	2022
JAPAN	6	66	72	904	12.56	5.71	1998	2022
CHINA	9	62	71	1632	22.99	5.63	1999	2022
UK	7	63	70	1419	20.27	5.55	1998	2022
TURKEY	10	30	40	439	10.98	3.17	2011	2022
NETHERLANDS	1	34	35	1502	42.91	2.78	2001	2022
SWITZERLAND	3	29	32	652	20.38	2.54	2013	2022
SOUTH KOREA	2	29	31	751	24.23	2.46	1999	2022
AUSTRALIA	3	14	17	336	19.76	1.35	2010	2022
NORWAY	1	14	15	277	18.47	1.19	2008	2022
INDIA	4	10	14	95	6.79	1.11	2009	2022
SPAIN	1	12	13	379	29.15	1.03	2010	2021
POLAND	2	10	12	117	9.75	0.95	1999	2022
BRAZIL	2	10	12	182	15.17	0.95	2015	2022
TAIWAN	3	9	12	134	11.17	0.95	2002	2022
AUSTRIA	1	10	11	278	25.27	0.87	2005	2022

Collaboration Pattern

**Figure 4:**
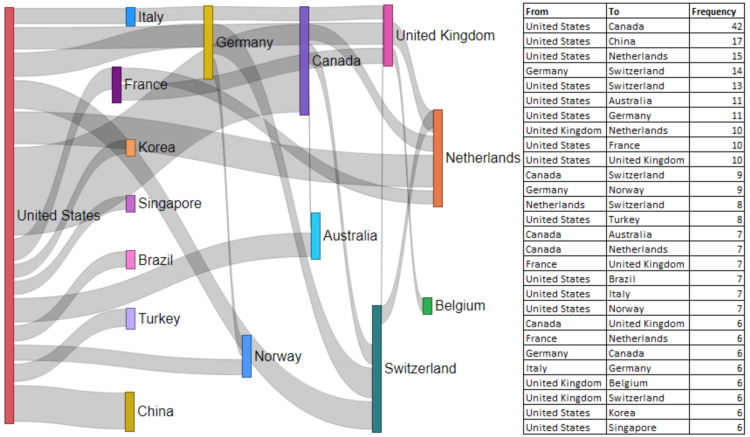
Study collaboration between different countries in the field of re-irradiation.

Figure 4 illustrates the collaboration network between countries in the field of re-irradiation using stereotactic radiotherapy. USA and Canada had the highest collaboration in terms of publications (n=42) followed by USA and China (n=17) and USA and Netherlands (n=15). The United States collaborated with 14 countries for publications, followed by Canada with four countries and Germany with three countries.

Three-Factor Analysis (Countries, Keywords, and Journals)

**Figure 5:**
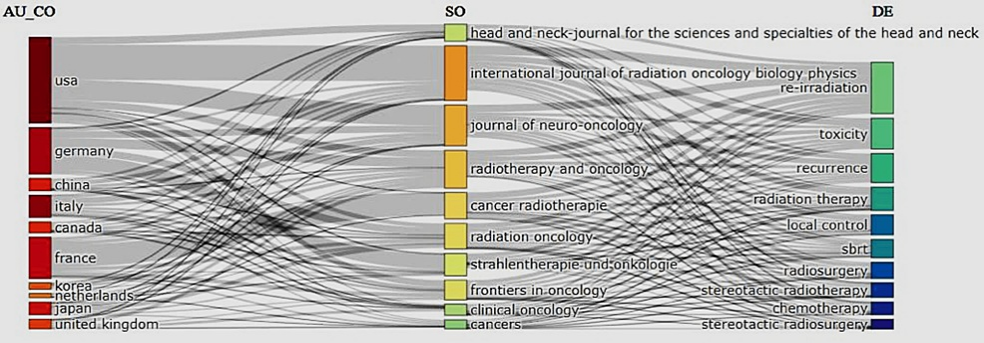
Three-factor analysis of the relationship between keywords (right), journals (center), and countries (left).

Figure 5 shows a three-factor analysis of the relationships between countries, keywords, and journals. We show that the top five countries (USA, Germany, France, Italy, and Japan) publish their reirradiation literature primarily using four main keywords: reirradiation, toxicity, recurrence, and radiotherapy. These countries and keywords have strong relationships with three journals (International Journal of Radio Oncology Biology Physics, Journal of Neuro-Oncology, and Radiotherapy and Oncology). The leading positions of developed countries in medicine and medical journals with great influence in this field make these countries the most important sources of publications and publishing institutions. 

**Figure 6:**
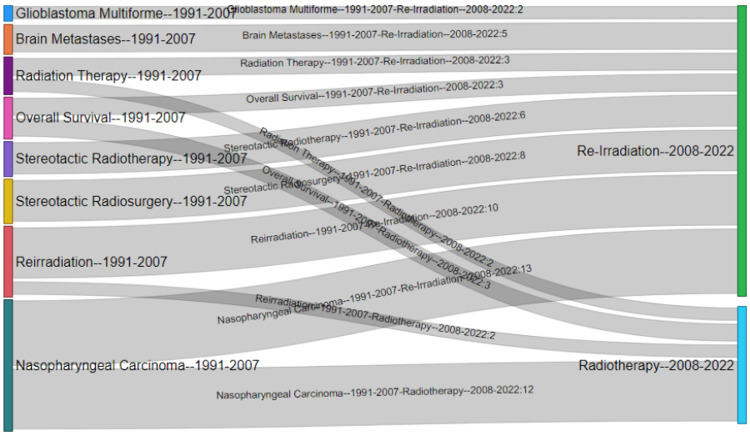
Thematic evolution map of keywords from 1991 to 2022 for re-irradiation using stereotactic radiotherapy.

Figure 6 visually presents the progression of keywords across two distinct time periods, namely 1991-2008 and 2008-2022. Notably, keywords such as "overall survival," "brain metastases," "stereotactic radiotherapy," and "stereotactic radiosurgery" emerged as a prevalent theme during the initial phase from 1990 to 2008. Subsequently, in the period from 2008 to 2022, these keywords merged with the themes of "re-irradiation" and "radiotherapy," demonstrating an evolving trend in the research landscape.

Highly Cited Articles

Table 4 lists the top 10 publications based on the overall number of citations and the number of citations rate each year [19-28]. Among them, three articles referred to spinal metastasis [19,21,28], and three articles referred to gliomas [22-24]. Out of the top 10 articles with the highest number of citations, six were published in the International Journal of Radiation Oncology Biology Physics, which has an impact factor of 7.04.

**Table 4:** List of 10 articles with the highest citation rate in the area of re-irradiation. Ref - reference; TI – title; TC – total citations; CPY – citations rate per year

	Ref	TI	TC	CPY
1	Kirkpatrick Jp, 2010, Int J Radiat Oncol Biol Phys [19]	Radiation dose-volume effects in the spinal cord	317	24.38
2	Catton C, 1996, Radiother Oncol [20]	Chordoma: long-term follow-up after radical photon irradiation	280	10.37
3	Laufer I, 2013, J Neurosurg-Spine [21]	Local disease control for spinal metastases following "separation surgery" and adjuvant hypofractionated or high-dose single-fraction stereotactic radiosurgery: outcome analysis in 186 patients	279	27.9
4	Grosu Al, 2005, Int J Radiat Oncol Biol Phys [22]	Reirradiation of recurrent high-grade gliomas using amino acid PET (SPECT)/CT/MRI image fusion to determine gross tumor volume for stereotactic fractionated radiotherapy	261	14.5
5	Combs Se, 2005, J Clin Oncol [23]	Efficacy of fractionated stereotactic reirradiation in recurrent gliomas: long-term results in 172 patients treated in a single institution	229	12.72
6	Fogh Se, 2010, J Clin Oncol [24]	Hypofractionated stereotactic radiation therapy: an effective therapy for recurrent high-grade gliomas	224	17.23
7	Schulz-Ertner D, 2004, Int J Radiat Oncol Biol Phys [25]	Results of carbon ion radiotherapy in 152 patients	212	11.16
8	Jereczek-Fossa Ba, 2012, Int J Radiat Oncol Biol Phys [26]	Robotic image-guided stereotactic radiotherapy, for isolated recurrent primary, lymph node or metastatic prostate cancer	193	17.55
9	Mayer R, 2008, Int J Radiat Oncol Biol Phys [27]	Reirradiation tolerance of the human brain	177	11.8
10	Sahgal A, 2009, Int J Radiat Oncol Biol Phys [28]	Stereotactic body radiotherapy is effective salvage therapy for patients with prior radiation of spinal metastases	160	11.43

Discussion

Re-irradiation for loco-regional recurrences is becoming more common as cancer patients live longer [29]. This bibliometric analysis demonstrates that research on re-irradiation using stereotactic radiotherapy has steadily increased over the past three decades, with a marked increase in the last decade. This may enable a paradigm shift in the critical clinical setting where alternative treatment options are limited. Recent advances in treatment techniques, such as the ability to account for dose accumulation, deformable registration, intensity modulation, and image guidance make it a more compelling option [30]. Furthermore, the availability of new techniques such as stereotactic radiotherapy, image-guided radiosurgery, and brachytherapy have led many clinicians to consider re-irradiation for more sites. This is because these techniques can preserve critical and slow-reacting tissues. The most important factor in planning is that these techniques can be used to determine the dose of various tissues within the irradiated volume. 

One of the most prevalent side effects for cancer patients is brain metastases and using stereotactic radiosurgery has long been regarded as the gold standard of care with sufficient literature support [31-33]. Over the course of the last decade, the utilization of stereotactic ablation radiotherapy (SABR or SBRT) has experienced a notable expansion. Initially, SABR was restricted to early-stage primary lung cancer, but as part of the results of several clinical studies, its use is now extended to non-pulmonary primary disease sites as well as oligometastatic settings. There is evidence that SABR is well tolerated and achieves high local control rates in some settings [34]. Many studies have examined SBRT for spinal metastases in pre-irradiated areas over the past decade [35-37]. In all studies, image-guided radiosurgery, intensity modulated radiotherapy (IMRT), or robotic stereotactic radiosurgery systems were used for highly conformal dose distributions.

The term bibliometrics simply refers to a set of methods (statistical and mathematical) for measuring and analyzing the quantity and quality of publications [18]. This analysis uses the simplest method available to assess a researcher’s productivity (the number of articles published in WoSCC). While it is a very straightforward indicator that is easy to calculate, one should be extremely cautious when comparing different groups based on it. Although the number of publications reflects productivity, it does not address the quality of the articles [18]. In addition to productivity, performance indicators can be used to identify the quality of the publication. An impact factor derived from the Journal Citation Index was used to measure quality in this study. As is well known, a journal's impact factor and an article's citation rate are the key metrics for assessing their respective impact [38]. To our knowledge, no literature analysis on reirradiation has been performed. As a result, this study is the first to examine the bibliometric characteristics and evaluate the citation patterns of radiation studies at various sites in order to present an impact indicator in this sector.

Limitations

Identifying publications was limited to the Web of Science search engine, so publications from other databases or in languages other than English may have been missed, leading to bias in citation statistics. Several variables affect citation counts, including the author, the field of study, and time since publication. For instance, early publications typically had more citations. Since the key articles analyzed with the highest number of citations were published before 2013, some new publications may have been overlooked. The study used annual citation counts to compensate for the effect of publication time on the most cited articles.

## Conclusions

To our knowledge, this is the latest bibliometric analysis of the most cited studies on re-irradiation. Our results provide insight into the historical development of reirradiation and important advances in its application to cancer therapy. Re-irradiation management presently relies on a multidisciplinary integration of sophisticated imaging, stereotactic therapy delivery, toxicity, and biological dose buildup. The main areas of interest have changed over time and are now based on a multidisciplinary approach that integrates advanced imaging techniques like deformable registration, stereotactic treatment delivery, the toxicity of organs at risk from re-irradiation, quality of life, sustainability of treatments, overall efficacy and effectiveness of treatments and treatment outcomes. This study helps researchers identify the most influential research on reirradiation and current research priorities. 
